# Expression Patterns of Circular RNAs in High Quality and Poor Quality Human Spermatozoa

**DOI:** 10.3389/fendo.2019.00435

**Published:** 2019-07-03

**Authors:** Teresa Chioccarelli, Francesco Manfrevola, Bruno Ferraro, Carolina Sellitto, Gilda Cobellis, Marina Migliaccio, Silvia Fasano, Riccardo Pierantoni, Rosanna Chianese

**Affiliations:** ^1^Dipartimento di Medicina Sperimentale, Università degli Studi della Campania L. Vanvitelli, Naples, Italy; ^2^UOSD di Fisiopatologia della Riproduzione, Presidio Ospedaliero di Marcianise, Caserta, Italy

**Keywords:** circRNAs, spermatozoa, miRNAs, sperm quality, fertilization, embryo development

## Abstract

Circular RNAs (circRNAs) are expressed in human testis and seminal plasma. Until today, there is missing information about a possible payload of circRNAs in human spermatozoa (SPZ). With this in mind, we carried out a circRNA microarray identifying a total of 10.726 transcripts, 28% novel based and 84.6% with exonic structure; their potential contribution in molecular pathways was evaluated by KEGG analysis. Whether circRNAs may be related to SPZ quality was speculated evaluating two different populations of SPZ (A SPZ = good quality, B SPZ = low quality), separated on the basis of morphology and motility parameters, by Percoll gradient. Thus, 148 differentially expressed (DE)-circRNAs were identified and the expression of selected specific SPZ-derived circRNAs was evaluated in SPZ head/tail-enriched preparations, to check the preservation of these molecules during SPZ maturation and their transfer into oocyte during fertilization. Lastly, circRNA/miRNA/mRNA network was built by bioinformatics approach.

## Introduction

Research on circular RNA (circRNA) biogenesis, spatial distribution, and physiological roles in the cells is at the beginning.

CircRNAs are covalently closed RNAs produced by back-splicing reactions (1). Once considered splicing by-products or artifacts, they represent nowadays a rising star of the RNA family, because of their resistance to nucleic acid exonucleases and their great potency as gene expression regulators (2–4). According to their structure, circRNAs can be classified into three categories: exonic, intronic (ciRNAs), and exon-intron (EIciRNAs), with the first ones preferentially located in the cytoplasm, while ciRNAs and EIciRNAs are located in the nucleus (5). In addition, “antisense” are circRNAs having their gene locus overlapped with the linear RNAs, but transcribed from the opposite strand; “sense overlapping” are circRNAs transcribed from the same gene locus as the linear counterpart, but not classified as exonic or intronic; “intergenic” represent circRNAs located outside known gene locus.

Their main role is to modulate microRNAs (miRNAs) and protein availability working as sponges (6); this tethering activity inhibits miRNAs, protecting their mRNA targets from degradation. For this reason circRNAs are part of the competitive endogenous RNA (ceRNA) network (ceRNET). Besides tethering activity, circRNAs also regulate transcription of their host genes (7) and splicing of their cognate mRNAs (8).

In first studies circRNAs were studied in cancer and other human diseases (9, 10); only recently, they have been identified to play a role in physiological conditions in several cell types, tissues, and organisms (11). Brain is the main site of circRNA production (12); here, circRNAs are stored, dynamically distributed among neuronal compartments and with age-dependent floating pattern. Interestingly, testis—as brain—shows similar impressive amount of circRNAs (13), suggesting their potential contribution to spermatogenesis. In rodents, circRNA pattern has been analyzed in mitotic, meiotic and post-meiotic germ cells (14) and correlated to sexual development (15). Similarly to testis, human ovary expresses a large number of circRNAs with an aging-dependent modulation (16). Considering circRNA production in both testis and ovary, a potential involvement of these non-coding RNAs in embryo development appears plausible. In this regard, a complete landscape of embryo transcriptome, including circRNAs, has been painted and it is generally accepted to be of maternal origin (17). Concerning the paternal contribution in term of circRNAs to the embryo is still an unexplored field.

During their post-testicular maturation, SPZ undergo a dramatic switch in their RNA payload, enriching themselves of epididymal epithelial cell products through epididymosomes (18). CircRNAs—together with miRNAs and tRNA fragments—may take part to the repertoire of non-coding RNAs used by SPZ to vehicle paternal experiences to the offspring and, therefore, may represent important instrument for trans-generational epigenetic inheritance.

Very recently, we identified and functionally characterized—for the first time—a circRNA, *circNAPEPLD*, in both human and murine SPZ; we also suggested that SPZ-derived *circNAPEPLD* may represent a paternal contribution to the zygote (19). Thus, we extended our knowledge by profiling circRNAs in human SPZ by circRNA microarray and analyzing all characteristics concerning these molecules by bioinformatics analysis. Then, we asked whether (a) circRNAs were correlated to SPZ quality, (b) SPZ-dependent circRNA payload was preserved during SPZ maturation and transferred into oocyte during fertilization. Both hypotheses were speculated (a) carrying out circRNA microarray on two different SPZ populations obtained in healthy normo-zoospermic volunteer donors by Percoll gradient separation, in order to identify differentially expressed (DE)-circRNAs as a function of SPZ quality, evaluated on the basis of morphology and motility parameters (20); (b) investigating circRNA subcellular localization in SPZ head and tail-enriched preparations. Lastly, circRNA-miRNA-mRNA networks related to sperm quality and embryo development were also constructed by using bioinformatics tools.

## Materials and Methods

### Human Semen Samples

Semen samples were obtained by healthy normo-zoospermic volunteer donors (*n* = 30) with age ranging from 25 to 45. After 3–5 days of sexual abstinence, semen samples were produced by masturbation and collected in sterile sample containers, which were delivered to the laboratory within 1 h of ejaculation. The sperm samples were allowed to liquefy for 30 min at 37°C and assessed in volume, concentration and motility according to World Health Organization (21) reference criteria by using a computer-assisted sperm analysis (CASA) technology. Successively, sperm samples were purified on a Percoll density gradient.

### Ethical Approval

This study was carried out in accordance with WHO guidelines. Sperm collection was preceded by written informed consent of participants, in accordance with the Declaration of Helsinki. Subjects were interviewed to better understand their area of origin, their eating habits as well as their lifestyles.

This study was carried out in accordance with WHO guidelines, approved by Regione Campania—acting as Azienda Sanitaria Locale (ASL) Caserta—prior agreement of ethics committee (n. 1353 del 27.10.2017).

### SPZ Isolation by Percoll Density Gradient Centrifugation

Purification of human SPZ was achieved using a 40 and 80% discontinuous Percoll (GE Healthcare, Castle Hill, Australia) centrifugation gradient. For this procedure, Percoll (90 ml) was supplemented with 10 ml of Dulbecco phosphate buffered saline (PBS) 10-fold concentrated solution (Lonza, Basel, Switzerland). The resulting solution (considered to be 100% Percoll) was further diluted with PBS 1x to give 40 and 80% Percoll solutions. The pH was equilibrated to 7.4. The gradient was prepared by placing 1 ml of each 40/80% solutions in a conical plastic tube 30 mm in diameter. The human semen sample (1 ml) was loaded at the top of the gradient and centrifuged at 300 × *g* for 20 min. Following centrifugation, the seminal plasma was removed and discarded and each fraction was collected by aspiration, beginning from the upper layer. In particular, purified viable and motile SPZ were recovered from the base of the 80% Percoll fraction (“A SPZ”) while abnormal SPZ were recovered from 40% Percoll fraction (“B SPZ”). A and B SPZ pellets were washed once with 10 ml of PBS to remove the Percoll, followed by centrifugation at 500 × *g* for 15 min, and were analyzed in terms of motility and morphology through CASA technology. To do this, we took advantage from Sperm Class Analyzer (SCA) software that records the number of motile/immotile SPZ, with an accurate counting, and it determines SPZ trajectory and velocity. SPZ morphology was, instead, evaluated by staining an aliquot of A and B pellets with SpermBlue, following the manufacturer's instructions.

In addition, both SPZ fractions were evaluated under light microscopy to estimate possible contaminations by somatic cells; thus, they were treated with Somatic Cell Lysis Buffer [SCLB; (22)] consisting of 0.1% SDS, 0.5% Triton X-100 in DEPC-H_2_O. Lysis proceeded by incubating cells on ice for 30 min. Microscope examination was used to verify the elimination of somatic cells. If somatic cells persisted, SCLB treatment was repeated by centrifuging the sample and resuspending the pellet in fresh SCLB. Finally, when somatic cells were absent, sample was centrifuged at 200 × *g* for 15 min at 4°C; SPZ, contained in the pellet, were counted by CASA and frozen at −80°C prior to processing for total RNA isolation.

### Separation of Sperm Into Head- and Tail-Enriched Fractions

The sperm pellets of fraction A from 5 healthy normo-zoospermic volunteer donors were separately resuspended in 5 ml Mitochondrial Wash Buffer (0.25 M sucrose, 0.01 M Tris-HCl, 0.001 M EDTA, pH 7.6) and sonicated at 4°C, 3 × 20 s at 20 Hz, using Soniprep150 Ultrasonic Disintegrator (SANYO Electric Co. Ltd., Japan). The sonicates were layered over a discontinuous sucrose gradient containing 10 ml 0.9 M sucrose and 15 ml 2.0 M sucrose, prepared in Buffer B (0.01 M Tris-HCl, 0.001 MEDTA, pH 7.6). The gradients were centrifuged at 10,000 rpm in a SW28 rotor in Beckman Optima L-90K Ultracentrifuge (Beckman Coulter, USA) at 10°C for 30 min. Sperm tails were collected from the interface of 0.9 M sucrose and 2.0 M sucrose, and the heads from the bottom of the tube. Head and tail purity was firstly examined by microscopic analysis; both preparations were then washed with 5 ml Buffer B, centrifuged at 10,000 rpm, 10°C for 30 min and finally frozen and stored at −80°C for later analysis.

### Total RNA Preparation

Total RNA was extracted from human SPZ using Trizol® Reagent (Invitrogen Life Technologies, Paisley, UK) following the manufacturer's instructions. In brief, the sample was homogenized in Trizol Reagent (1 ml Trizol® Reagent/5–10 × 10^6^ sperm cells); after homogenization, the sample was incubated for 5 min at 20°C to permit the complete dissociation of nucleoprotein complexes. Then 0.2 ml chloroform/ml Trizol® Reagent was added and the sample centrifuged at 12,000 × *g* for 15 min at 4°C. The aqueous phase was transferred to a fresh tube and total RNA was precipitated by mixing with isopropyl alcohol (0.5 ml/ml Trizol® Reagent) and 1 μl glycogen (20 mg/ml) to promote the precipitation of small size RNAs. After centrifugation at 12,000 × *g* for 10 min at 4°C, the RNA pellet was washed with 75% ethanol, centrifuged at 7,500 × *g* for 10 min at 4°C and dissolved in an appropriate volume of DEPC treated water. The quantity (ng/ml) and purity (260/280 and 260/230 ratios) of total RNAs were assessed with a NanoDrop 2000 spectrophotometer (Thermo, Waltham, MA, USA). To remove potential contamination of genomic DNA, RNA aliquots (10 μg) were treated with 2U DNase I (RNase-free DNase I, Ambion, Thermo Fisher Scientific, Massachusetts, USA) according to the manufacturer's recommendations. Finally, total RNAs were digested with RNase R (Ribonuclease R, *E. coli*, Cat. No. RNR07250, Epicenter, Madison, Wisconsin, USA) 10 U enzyme/1 μg RNA, at 37°C for 10 min to remove linear RNAs and enrich samples of circRNAs, followed by heat inactivation at 95°C for 3 min. The RNAs were then preserved at −80°C until the next step.

### circRNA Microarray

The sample preparation and microarray hybridization were performed according to the Arraystar's standard protocols (Arraystar, Rockville, MD). Briefly, the enriched circRNAs were amplified and transcribed into fluorescent cRNA utilizing a random priming method according to Arraystar Super RNA Labeling protocol (Arraystar, Inc.). The labeled cRNAs were purified by RNeasy Mini Kit (Qiagen). The concentration and specific activity of the labeled cRNAs (pmol Cy3/μg cRNA) were measured by NanoDrop ND-1000. One microgram of each labeled cRNA was fragmented by adding 5 μl 10 × Blocking Agent and 1 μl of 25 × Fragmentation Buffer, then heated the mixture at 60°C for 30 min, finally 25 μl 2 × Hybridization buffer was added to dilute the labeled cRNA. Fifty microliter of hybridization solution was dispensed into the gasket slide and assembled to the circRNA expression microarray slide. The slides were incubated for 17 h at 65°C in an Agilent Hybridization Oven. The hybridized arrays were washed, fixed and scanned using the Agilent Scanner G2505C (Agilent Technologies, CA).

### Expression Profiling Data

Agilent Feature Extraction software (version 11.0.1.1) was adopted to analyze acquired array images. Quantile normalization of raw data and subsequent data processing were performed using the R software package. After quantile normalization of the raw data, low intensity filtering was performed, and the circRNAs that at least 3 out of 6 samples have flags in “P” or “M” (“All Targets Value”) were retained for further analyses.

### Differential Expression Analysis

Two groups of circRNAs were identified in A and in B SPZ, respectively, by using the R software package and conveniently compared by *t*-test in order to select DE-circRNAs, with *p* < 0.05 and fold changes>1.5. Since the comparison between A and B SPZ, we used along the text the expression “B compared to A SPZ” to indicate statistically significant DE-circRNAs as showed by volcano plot filtering. DE-circRNAs among samples were identified through Fold Change filtering. Hierarchical clustering was performed to show the distinguishable circRNAs expression pattern among samples.

### Functional Annotation for circRNA/miRNA and Target miRNA Interaction

The circRNA/miRNA interaction was predicted with Arraystar's miRNA target prediction software based on both TargetScan (23) and MiRanda online analytical software (24); such an analysis was performed for all DE-circRNAs.

For functional annotation, all parental genes of the DE-circRNAs were subjected to KEGG (www.genome.jp/kegg) pathway enrichment analyses using DAVID Bioinformatics Resources 6.8 (david.ncifcrf.gov/home.jsp). The *p*-value was calculated using a hypergeometric test and corrected by Benjamini-Hochberg adjustment. We regarded the Fold Enrichment as the enrichment score that indicated the significance of correlation. Validated or predicted targets of miRNAs were retrieved by Diana TarBase 8.0 (http://www.microrna.gr/tarbase); circRNA/miRNA/Target network was built and visualized by using Bisogenet plug-in of Cytoscape (https://cytoscape.org/).

### PCR Primer Design

Primers to validate and amplify selected circRNAs in human SPZ samples were designed through the online tool Primer-BLAST (http://www.ncbi.nlm.nih.gov/tools/primer-blast/). In order to make primers specific for the circular isoforms, we designed primers spanning the back-splicing junction. We also designed specific primers for the housekeeping gene used for normalization: *GAPDH* (glyceraldehyde 3-phosphate dehydrogenase). Primers for human genes are shown in Table 1.

**Table 1:** Primer Sequence and Annealing Temperature for circRNAs.

**Gene primers**	**Sequences 5^**′**^-3^**′**^**	**Tm (^**°**^C)**
*circANKLE2 S* *circANKLE2 AS*	ACCACGTCTGGCAGAAGATTT TTTCCGGGTCCGACTTCTTG	55
*circARIH1 S* *circARIH1 AS*	CAGTTATGCGCCTGATCACAG CCCAATTGAAGTGGCTAAGGAGTA	55
*circATF7IP S* *circATF7IP AS*	GCAGGCACAGTGAGACAGA GGTTGGGTGGATGCTGTATTC	58
*circCNOT6L S* *circCNOT6L AS*	GCCTTATGAACTTGGTCGGCT TTCTGCGAGGATCTGGAGGAT	56
*circDNAJB6 S* *circDNAJB6 AS*	TGACTTCTTTGGACCCATTCCA TGTCTCTGCACGCCTAGAAC	56
*circEIF2C2 S* *circEIF2C2 AS*	CGGGATCACCTTCATCGTGG GTATGATCTCCTGCCGGTGC	58
*circGAPDH S* *circGAPDH AS*	TGCACCACCAACTGCTTAGC GGCATGGACTGTGGTCATGAG	58
*circGPBP1L1 S* *circGPBP1L1 AS*	CACCGACAGGAAGAGTGAGTT ATGGCTGATGCTGTTTGCCA	58
*circGRB10 S* *circGRB10 AS*	TCACGCCGGGTTCTTTACCTC TAGAAGGAGGATGTTTGTTCCCTG	58
*circGUSBP1 S* *circGUSBP1 AS*	CAGAGCGTGTATGGAGTGGA GGTCACAAAGGTCACAGGCT	56
*circHACE1 S* *circHACE1 AS*	GTGAATGGGCGGACAGAACT CCACCGATCCACAACGTCTT	55
*circJA760600 S* *circJA760600 AS*	GGGCTTTGGTGAGGGAGGTA ATACTACCGTATGGCCCACC	55
*circKIF24 S* *circKIF24 AS*	TATTGACTTGGCTGGCAGTGA TGCAAAGAGCAGCCAGTAGA	55
*circKIF2C S* *circKIF2C AS*	CAACCCTGCTACCGGAAGTT CTTGCTGTGAACCTTCCCATT	56
*circL3MBTL4 S* *circL3MBTL4 AS*	GGTCTTGGGAGTGGTACTTGA CCTTGGCAGTGGTTTTATGGTC	58
*circLZIC S* *circLZIC AS*	ACAGACCTTGGCTATTCAGGC AACCTTGTCCGAAGCTGACC	56
*circMLLT3 S* *circMLLT3 AS*	GGCAGCGATAGTGAAAGCAG TGGGTTGTTCAGACTGGTTGT	56
*circMTND5 S* *circMTND5 AS*	GCAGCCTAGCATTAGCAGGAAT GGGAGGTTGAAGTGAGAGGTA	56
*circPTTG1IP S* *circPTTG1IP AS*	AGGAACGGAGAGCAGAGATGA TTTACAAAGGGAAGCCGGTG	55
*circRASA3 S* *circRASA3 AS*	GCAGAAGTACCACAACAGGGA TTTGGCTTCACCTGCACTTC	56
*circSEC24A S* *circSEC24A AS*	TGCTTCCTGGCAACACTAGAA TGAGGTGGTCGTAACTTCAATATC	55
*circSENP6 S* *circSENP6 AS*	GCCTGTAATTGAAAGAATACCCACC ACCTTGGAGCCGACTTAACC	55
*circUBA2 S* *circUBA2 AS*	CAAGCTGAAGATGCTGCCAAA AACTGTCTGTTGAGGTTGCTT	55
*circUBXN7 S* *circUBXN7 AS*	ACCATTATTTGGTGGTGCAAGT ACTGGGCTCTTCAGCGATTC	58
*circVMP1 S* *circVMP1 AS*	TCGGTACAGCAATCGGAGAG TGTGGACCTTGTGCAGACTC	55
*circWDR66 S* *circWDR66 AS*	GGCCGTTTCCTATGATGGCT GGCGTGGCCCTTAAGGTGAT	58
*circZFAT S* *circZFAT AS*	GAACATCCTGCAGCAGATCATTG GGACACTTCAAACCACCTGC	55
*circZMYND11 S* *circZMYND11 AS*	GCCCAGTTTGCAGGCTAAAGA CAATGGCTGCCCAAAGATGC	55
*circZNF148 S* *circZNF148 AS*	CACTTAATGTCCCTGCAGTTATAC GCCAGACACTCCACCCATT	58

### circRNA Expression Analysis by One-Step Evagreen qRT-PCR

We investigated circRNA expression through One-Step Evagreen qRT-PCR reaction by using a kit containing qRT-PCR enzyme mix and an Evagreen qPCR Mastermix (Applied Biological Materials Inc.), according to the manufacturer's instructions. All reactions were performed by using 50 ng of total RNA on a CFX-96 Real Time PCR System (Biorad). Assays were carried out in triplicates and included a melting curve analysis for which all samples displayed single peaks for each primer pairs. A negative control, without RNA, was also included. RNA expression was evaluated through CFX Manager software (Biorad); normalization was performed using *GAPDH* as housekeeping gene. Normalized fold expression of circRNAs was calculated by applying the 2^−ΔΔ*Ct*^ method.

### Statistical Analysis

All the data are expressed as the mean ± SEM for triplicate independent measurements. Student's t test was used to assess the differences between experimental groups. Differences with *p* < 0.05 were considered statistically significant.

## Results

### Overview of circRNA Expression in Human SPZ

**Figure 1:**
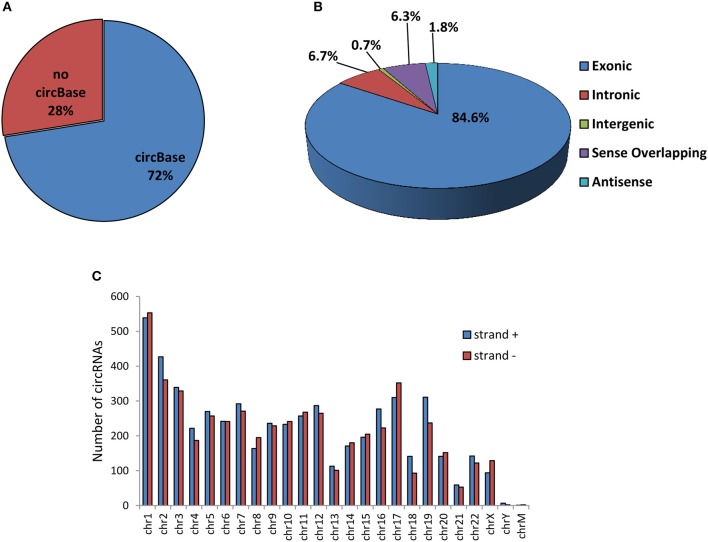
Overview of circRNA expression in human SPZ **(A)** The proportion of circRNAs from circBASE (www.circbase.org) and other databases/literatures on a total of 10,726 circRNAs identified. **(B)** The proportion of different types of circRNAs among all predicted circRNAs. **(C)** Chromosomal distribution of SPZ derived circRNAs, on strand + and strand –.

A total of 10,726 circRNAs were identified in human SPZ of which the majority (72%) are already present in the most common circRNA database (circBase), instead the 28% was novel based (Figure 1A). According to their structure, circRNA were distinguished in exonic, intronic, intergenic, sense overlapping, and antisense. Exonic circRNAs (84.6%) were the most abundant type of circRNAs in SPZ, while intergenic (0.7%) and antisense (1.8%) were less represented (Figure 1B). According to the location of their host genes, circRNAs were widely distributed across all chromosomes, including mitochondrial (M) chromosome, and on strand + or – (Figure 1C). However, the highest number of circRNAs was produced on chromosomes 1–3 and 17, while the lowest one was generated by chromosomes Y and M. Furthermore, circRNAs distributed on chromosomes 2, 16, 18, 19 predominantly derived from strand +; this profile was reversed for chromosomes 8 and 17 (Figure 1C).

### circRNA Expression in Fraction A Compared to B SPZ

**Figure 2:**
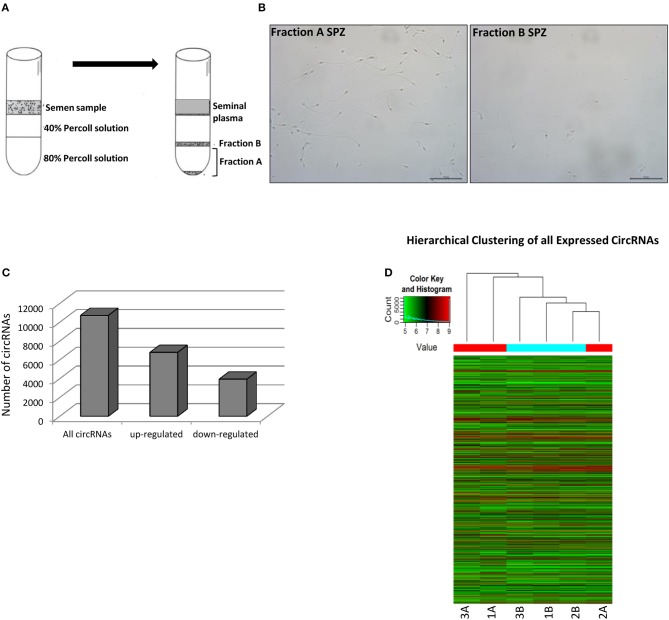
Expression of circRNAs in fraction A and B SPZ **(A)** Fractionation of human sperm by density gradient centrifugation. The lower and upper gradients (80 and 40%) were carefully layered and the semen sample located on the top. After centrifugation (see Materials and Methods section) clear seminal plasma was retained on the uppermost part of the gradient followed by a clear separation of white blood cells, debris and other cells. The immature, abnormal SPZ were recovered from 40% Percoll fraction (fraction B). Highly motile normal SPZ moved actively to the bottom of the gradient 80% and collected as a pellet (fraction A). **(B)** Fraction A and B SPZ isolated by semen using Percoll gradient visualized via optical microscopy. Scale bar 50 μm. **(C)** The distribution of up- and down-regulated circRNAs in fraction B compared to fraction A SPZ among all circRNAs. **(D)** Hierarchical clustering analysis of total circRNAs arranged samples into two groups, based on their expression levels; in detail, this analysis used different colors to represent the expression values of circRNAs detected in fraction A (*n* = 3, indicated as 1A, 2A, 3A) and B (*n* = 3, indicated as 1B, 2B, 3B) SPZ of normo-zoospermic volunteer donors.

In order to attribute to circRNAs a potential role in the control of sperm quality, we separated sperm, obtained from healthy normo-zoospermic volunteer donors (*n* = 3), in two different populations, on the basis of morphology and motility parameters, through a Percoll gradient separation (20). Fraction A SPZ was collected as pellet on the bottom of 80% Percoll solution; it contained high quality SPZ with good morphology and motility. Instead, fraction B, collected on the bottom of 40% Percoll solution, contained SPZ very poor in quality and not suitable for fertilization (Figure 2A). Quality of SPZ preparations was confirmed by microscopic examination after lysis treatment of possible somatic cell contaminations. Considering that the sperm was collected by normo-zoospermic donors, fraction B showed, physiologically, a lower number of SPZ if compared to fraction A (Figure 2B).

On the total of circRNAs, a larger number appeared up-regulated in B compared to A SPZ (Figure 2C). The result of hierarchical clustering clearly showed a distinguishable circRNA expression profiling between A SPZ (samples 1A, 2A, 3A) and B SPZ (samples 1B, 2B, 3B) (Figure 2D).

### Identification and Functional Annotation of DE-circRNAs in Fraction B Compared to A SPZ

**Figure 3:**
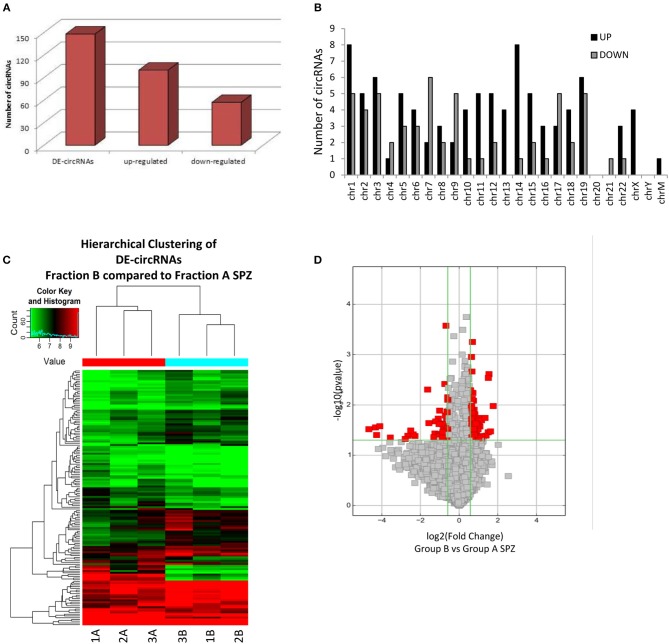
Differential expression of circRNAs between fraction A and B SPZ **(A)** The distribution of up- and down-regulated DE-circRNAs in fraction B compared to fraction A SPZ. **(B)** The distribution of up- and down-regulated DE-circRNAs in the human genome, in according to their host gene location. **(C)** Hierarchical clustering analysis of DE-circRNAs in A SPZ (samples 1A, 2A, 3A) and B SPZ (samples 1B, 2B, 3B); the expression values (Fold change >1.5, *p* < 0.05) were represented in different colors, indicating expression levels above and below the median expression level across all samples. **(D)** The volcano plot was constructed using Fold-Change and *p*-values; in detail, the values on X and Y axes are log2 (FC = Fold-Change) and –log10 (*p*-values), respectively. Red points in the volcano plot represent the DE-circRNAs with statistical significance.

By using more stringent filters such as fold change cut-off >1.5 and *p*-values cut-off <0.05, circRNA microarray analysis identified a total of 148 DE-circRNAs in fraction A (*n* = 3) and fraction B (*n* = 3) SPZ, consisting of 91 up-regulated and 57 down-regulated circRNAs in B compared to A SPZ, respectively (Figure 3A). According to their host gene location, the distribution of DE-circRNAs was analyzed in human genome (Figure 3B); interestingly, chromosomes 1, 10–16, 18, and 22 especially contained up-regulated circRNAs in B compared to A SPZ; such a profile was reversed on chromosomes 4, 7, 9, and 17. No DE-circRNA was detected on chromosomes 20 nor Y, instead chromosomes X and M only contained up-regulated circRNAs in B compared to A SPZ (Figure 3B). DE-circRNAs were clustered based on their expression levels in B and A SPZ as indicated by the hierarchical clustering plot (Figure 3C), and the volcano plot, this last one representing DE-circRNAs as red points (Figure 3D).

**Figure 4:**
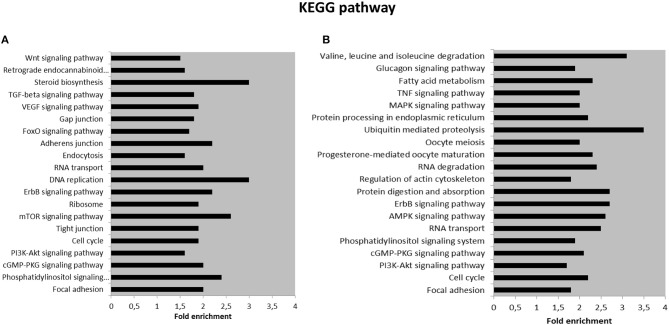
Kyoto Encyclopedia of Genes and Genomes (KEGG) pathway annotation of host genes **(A)** The Top 20 KEGG signaling pathway annotations in fraction B SPZ up-regulated circRNAs. **(B)** The Top 20 KEGG signaling pathway annotations in fraction B SPZ down-regulated circRNAs.

We further performed KEGG pathway analysis for the target genes of SPZ-derived circRNAs, as shown in Figures 4A,B. The Top 20 enriched KEGG signaling pathways were highly associated with steroid biosynthesis, DNA replication, mammalian target of rapamycin (mTOR) signaling pathway, RNA transport and degradation, cell cycle, and oocyte meiosis (Figures 4A,B).

### Functional Clustering of DE-circRNAs

**Figure 5:**
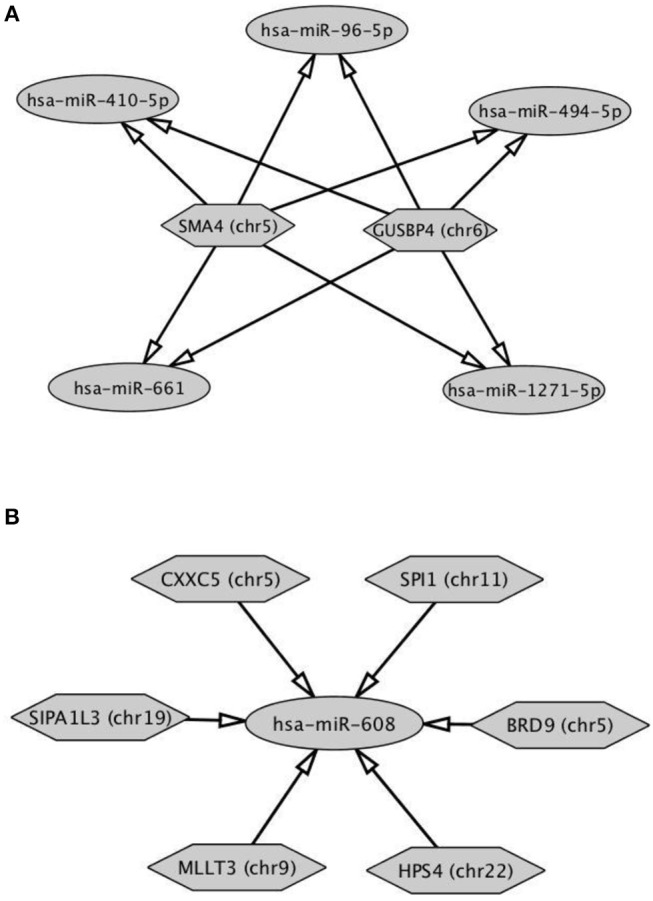
Functional clustering of DE-circRNA in B compared to A SPZ **(A)** Two circRNAs whose host genes are located on different chromosomes tether the same group of miRNAs. **(B)** More than two circRNAs show tethering activity toward the same miRNA as target. Networks were built using Cytoscape. Hexagonal and ovoid symbols represent circRNAs and miRNAs, respectively. The arrow indicates the tethering activity of circRNAs toward miRNAs.

The analysis of DE-circRNAs led us to hypothesize a particular way of circRNAs to work. CircRNAs (hexagonal symbols)—whose host genes are located on different chromosomes—showed tethering activity toward the same groups of miRNAs (ovoid symbols). An example of this functional behavior was built in Figure 5A and specifically involved *SMA4* and *GUSP4* circRNAs. Interestingly, this case was found exclusively in circRNAs down-regulated in B compared to A SPZ. On the other hand, more than two circRNAs, whose host genes are located on different chromosomes, were able to tether just the same miRNA. This second case was represented in both up- and down-regulated circRNAs in B compared to A SPZ (Figure 5B).

This analysis may suggest a functional clustering of SPZ-derived circRNAs.

### Experimental Validation of Predicted circRNAs in Human SPZ

Experimental validation of circRNA microarray results was preceded by a quality control of RNA extracted from SPZ. In fact, sperm obtained from epididymis often contain some somatic cells like epithelial cells, testicular germ cells and leukocytes. Thus, possible contamination by somatic cells (i.e., blood and epididymal cells) was excluded by analyzing the expression of non-sperm cell markers, such as *CD4* (biomarker of leukocytes) and *E-cadherin* (biomarker of epithelial cells) in samples; no significant signal was amplified by qPCR (data not shown). Because of controversial data regarding the choice of a good housekeeping gene for normalization in human SPZ, we randomly tested the expression level of *GAPDH* in 10 samples. Such an analysis revealed a stable abundance of *GAPDH* among samples suggesting to proceed for the normalization (data not shown).

**Figure 6:**
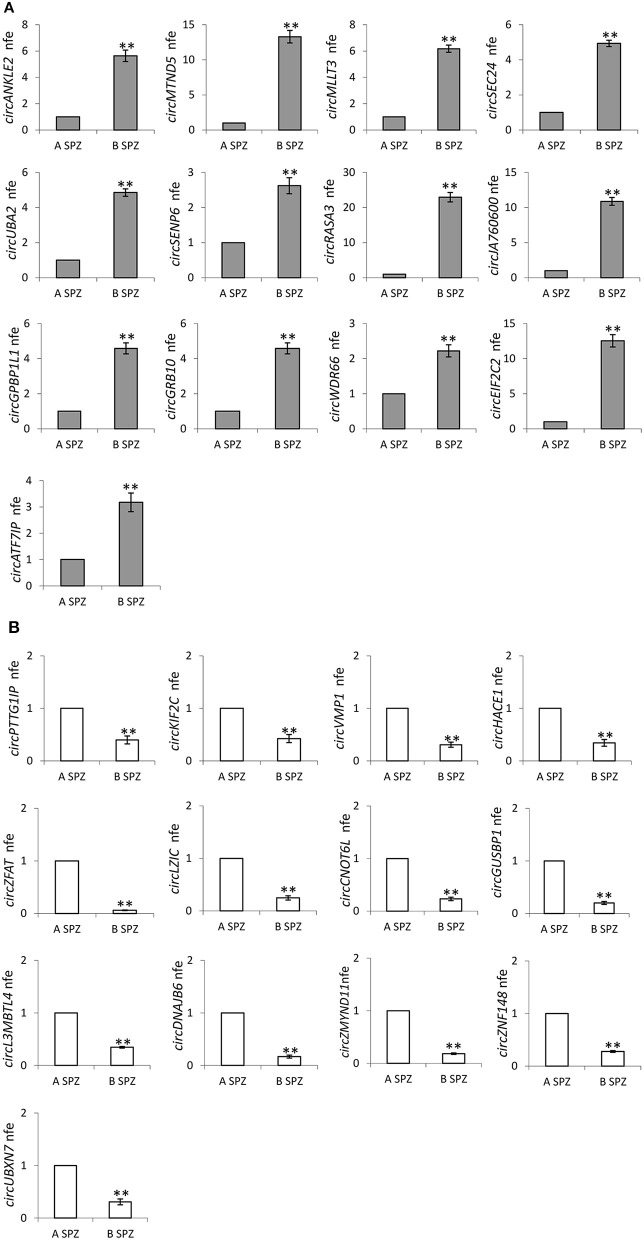
**(A)** Validation of the circRNA-microarray results up-regulated in B compared to A SPZ; **p < 0.01. **(B)** Validation of the circRNA-microarray results down-regulated in B compared to A SPZ; **p < 0.01.

Therefore, circRNAs—predicted in human SPZ and identified by circRNA microarray—were validated by qPCR, not randomly, rather we used a selective functional criterion: DE-circRNAs in human SPZ were carefully searched in Dang et al. circRNA database (17). After that, we selected circRNAs, surely expressed in SPZ and in some of the human embryonic developmental stages, but not in human oocytes, with the aim to suggest a potential paternal contribution in such an inheritance. Thus, we searched relative sequences in circBase and designed specific primers for circular isoforms, spanning the back-splicing junction to use for One-Step Evagreen qRT-PCR analysis (Figures 6A,B). The quality of Melting curves for each primer pair was carefully checked and only curves with single peaks were considered suitable for further analysis (Supplemental Figure 1).

QPCR analysis—performed in A SPZ (*n* = 22) and B SPZ (*n* = 22)—showed the expression of circRNAs up-regulated (*p* < 0.01; Figure 6A) and down-regulated (*p* < 0.01; Figure 6B) in B compared to A SPZ, respectively, thus to confirm circRNA microarray results.

### Subcellular Localization of Validated circRNAs in Human SPZ: Head or Tail?

**Figure 7:**
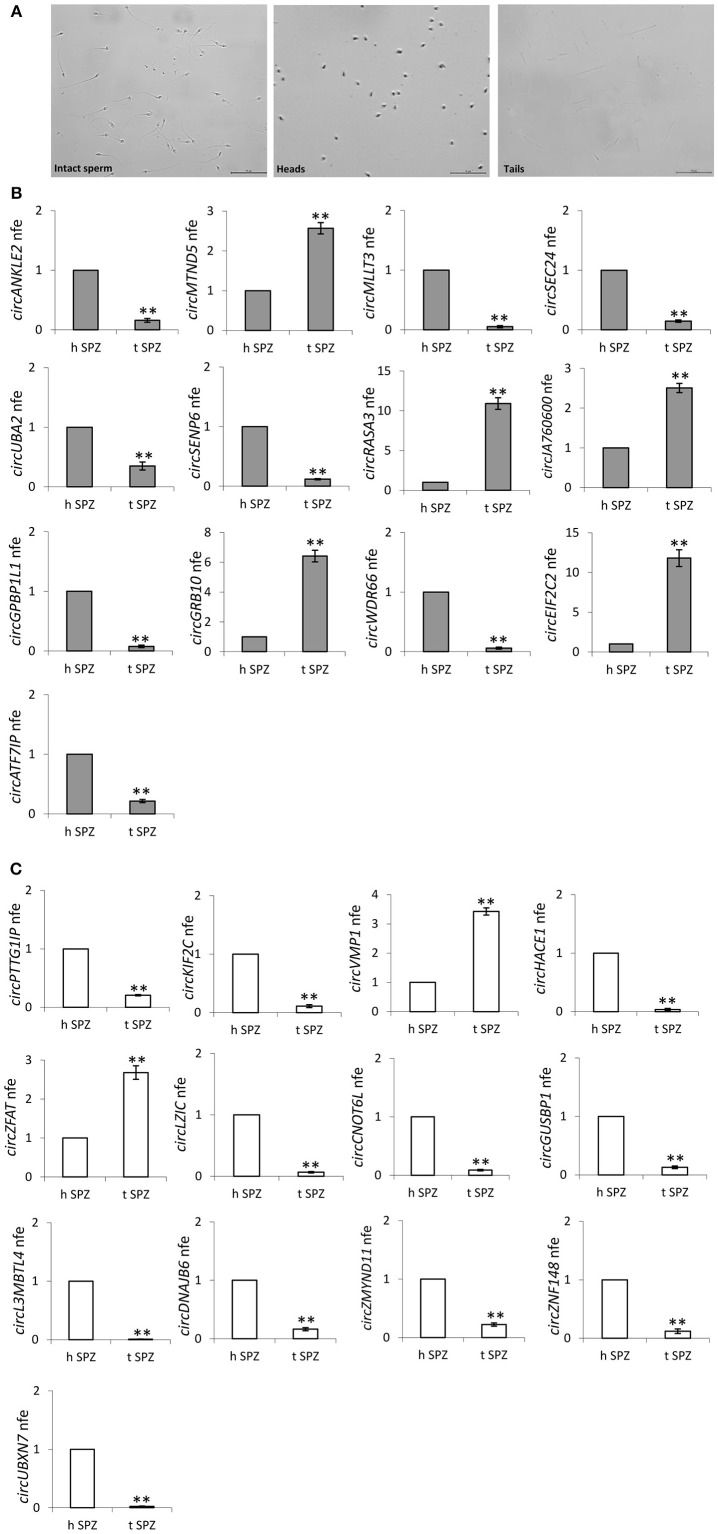
**(A,B)** Subcellular localization of sperm circRNAs **(A)** Separation of fraction A sperm into head- and tail-enriched fractions. Representative images of sperm showing intact sperm (left), along with head and tail-enriched fractions, as indicated. Scale bar 50 μm. **(B)** circRNAs up-regulated in B SPZ, differentially expressed in head and tail sperm; **p < 0.01. **(C)** CircRNAs down-regulated in B SPZ, differentially expressed in head and tail sperm; **p < 0.01.

Since our idea was that paternal circRNAs may contribute to egg fertilization and embryo development, we analyzed the subcellular localization of validated DE-circRNAs in A SPZ. In detail, SPZ collected from *n* = 5 normo-zoospermic volunteers were separated into heads and tails by sonication followed by sucrose gradient fractionation. Head- and tail- enriched fractions were evaluated by microscopic analysis and compared to intact sperm preparation (Figure 7A). The expression analysis of DE-circRNAs was, then, carried out by One-Step Evagreen qRT-PCR in both fractions. Interestingly, in both circRNA groups, up- and down-regulated in B compared to A SPZ (Figures 7B,C respectively), we discriminated circRNAs preferentially localized in the head rather than in the tail SPZ and viceversa (*p* < 0.01). Such a profile allowed us to focus our attention on circRNAs identified in head SPZ to suggest their potential role in the regulation of the first stages of embryogenesis. Accordingly, the top 5 of DE-circRNAs in both up- and down-regulated circRNA groups, were selected to build the circRNA-miRNA-mRNA interaction networks (ceRNET) presented in Supplemental Figure 2A (up-regulated circRNAs in B compared to A SPZ) and Supplemental Figure 2B (down-regulated circRNAs in B compared to A SPZ), respectively.

According to the results of bioinformatics prediction, several downstream mRNA targets are known to be associated with embryo development.

## Discussion

The ubiquitous expression of circRNAs in mouse and human cells (11, 25) and their tethering activity toward miRNAs and other RNAs make these molecules key members of the ceRNET, a powerful molecular system of gene expression regulation (6, 26). Furthermore, the large numbers of circRNAs in human tissues, especially in brain and testis (12), and their dynamic age-dependent modulation that mirrors sexual maturity (15) let us to hypothesize their role in regulating spermatogenesis and sperm quality.

CircRNAs have also been analyzed in human ovary (16), oocytes and pre-implantation embryos (17). Very recently, we demonstrated that human and murine SPZ preserve a circRNA, *circNAPEPLD*, with a tethering activity toward miRNAs, previously shown to be expressed in oocytes, and speculated the possibility that this molecule may be transmitted from SPZ to oocytes during fertilization (19). Starting from these data, we identified and annotated 10,726 circRNAs in human SPZ, 28% are novel based. As in human testis and ovary (13, 16), exonic circRNAs were the majority and widely scattered across all chromosomes. According to their host gene location and as already observed in testis (13), circRNAs were also distributed on M and sexual chromosomes, even if—differently from other chromosomes that generated hundreds of circRNAs—M and Y chromosomes generated only few circRNAs. By using KEGG pathway annotation, we discovered several important biological processes and pathways involving SPZ-derived circRNAs such as steroid biosynthesis, DNA replication, mTOR signaling pathway, RNA degradation and transport, cell cycle, oocyte meiosis.

In order to demonstrate that circRNAs may have a role in sperm quality control, we profiled circRNA expression pattern in two different populations of human SPZ, distinguished on the basis of morphology and motility parameters, through a Percoll gradient separation (20). As detailed in Materials and Methods section, the two sperm fractions were opportunely washed to eliminate Percoll contaminations, before evaluating motility and/or morphology parameters by CASA-System. Thus, A SPZ showed high quality in terms of morphology and motility, instead B SPZ were not suitable for fertilization. CircRNA microarray analysis—carried out on both separated SPZ populations—identified a total of 148 DE-circRNAs, consisting of 91 up-regulated and 57 down-regulated circRNAs in B compared to A SPZ, respectively. Genome analysis showed that chromosomes 20 and Y did not show any DE-circRNA, while chromosomes X and M only contained up-regulated circRNAs in B compared to A SPZ. Interestingly, DE-circRNAs show a peculiar behavior since (a) two DE-circRNAs have tethering activity toward the same group of miRNAs, (b) more than two DE-circRNAs have tethering activity toward just the same miRNA. What may be the physiological implication of such a phenomenon needs further investigation. Our hypothesis is that: case (a) circRNAs, different for chromosomal positioning of their host genes, exert their inhibitory action on the same group of miRNAs in order to induce the expression of down-stream mRNA targets implicated in the regulation of the same biological process. For example, all miRNAs described in Figure 5A have—as mRNA targets—members of zinc finger protein family or transcriptional/translational factors, all involved in embryo survival and development (27). This suggests the need of a more efficient control of some biological pathways through the involvement of more circRNAs. Case (b) more than two DE-circRNAs exert their inhibitory action on the same unique miRNA. This may represent a redundant mechanism to specifically regulate the expression of that miRNA. Furthermore, both case (a) and (b) led us to hypothesize that SPZ-derived circRNAs may work using functional clusters to guarantee a successful fertilization. Surely, this hypothesis needs to be experimentally confirmed in the future.

CircRNA microarray profiling was not randomly validated: we compared our database to Dang et al. circRNA database (17) and selected circRNAs, surely expressed in SPZ and in some of the human embryonic developmental stages, but not in human oocytes, for experimental validation. Such an analysis perfectly mirrored circRNA microarray results. Interestingly, JA760600, a piwi-RNA (piRNA, 28) was able to circularize and was up-regulated in B compared to A SPZ, together with MTND5, a mitochondrial encoded NADH dehydrogenase 5; both are involved in microsatellite instability events regarding mitochondrial genome (28). The presence of two circRNAs whose genes derive from mitochondrial genome in mature SPZ had attracted our interest, since in both human and animal models there is a maternal inheritance of mitochondria and mitochondrial DNA, guided by selective ubiquitin-dependent degradation of the sperm-borne mitochondria after fertilization (29). This result encouraged us to verify subcellular localization of validated circRNAs—both up- and down-regulated in B compared to A SPZ—in SPZ head and tail-enriched preparations. Interestingly, this experiment revealed circRNAs preferentially expressed in SPZ head preparation, thus suggesting their potential transmission from SPZ to oocyte during fertilization. Considering that circRNAs harbored several conserved miRNA binding sites (2), the construction of a ceRNET (Supplemental Figures 2A,B) is an useful instrument to shed light on predicted mRNA targets. Interestingly, *circMLLT3*—through has-miR-136-5p—controls *MTDH* and *PPP2R2A*, the first involved in normal embryo development (30), the second working as a key regulator of the timing of M-phase entry and exit in *Xenopus laevis* first embryonic mitosis (31). In *circSEC24A*-dependent network targets as *ABCA1, NPC1*, and *ADAM9* are involved in maternal-fetal cholesterol transfer and in developing brain (32–34); in addition, *circSEC24A* also regulates the expression of *Lin28B*, a well-known pluripotency-linked gene (35). *Lin28B* as well as *NOTCH1, SOX2, CBX4, ZEB2* are recurrent targets in several circRNA-dependent networks, known to be required for successful embryogenesis (36–39). Among mRNA targets, we also counted sirtuin family members, known to be involved in gamete biology (40), *SOX* and *HOX* genes known to be involved in the modulation of cell-extracellular matrix adhesion, implantation and embryogenesis (41–43). Several studies also suggest that embryo development and the “window” of implantation are critically influenced by endocannabinoid levels, especially anandamide, the main endocannabinoid. Low levels of fatty acid amide hydrolase (FAAH), the enzyme able to degrade anandamide, correspond with spontaneous miscarriage (44). FAAH expression is regulated by hsa-miR-6730-5p in *circWDR66*-dependent network. Several transcripts—included in the molecular signature of human trophectoderm cells—such as *GATA, DNMT*, stemness genes such as *NANOG* as well as Melanoma Antigen family (*MAGE*) genes, all are expressed during the initial embryo-trophectoderm transition in humans (45) as well as in down-regulated circRNA-dependent networks, together with *SMOC* genes (46), *Serpine1* (47) and Myo-inositol (MYO) genes (48, 49).

In conclusion, our study is the first circRNA profiling in human SPZ. Sequence structure and chromosome localization were features here described; the possibility of functional clustering as a way of circRNAs to work was also suggested. Differential pattern of expression of circRNAs correlated to sperm quality and circRNA subcellular localization in SPZ were also evaluated. Whether circRNAs may be used for therapeutic treatments in male infertility or pointed as key molecules contributing to paternal trans-generational epigenetic inheritance and/or in which step of embryogenesis their activity may be essential, all these hypotheses still remain unanswered questions encouraging future investigations.

## Data Availability

The dataset for this manuscript are not publicly available with respect for individual privacy of participants. Requests to access the datasets should be directed to prof. Riccardo Pierantoni, riccardo.pierantoni@unicampania.it.

## Author Contributions

RC, TC, FM, GC, and RP designed the study. FM, MM, TC, CS, and RC performed the experiments. TC and RC drafted the manuscript and prepared figures. BF, GC, SF, RP, and RC critically revised the manuscript. All the authors approved the final version of the manuscript.

### Conflict of Interest Statement

The authors declare that the research was conducted in the absence of any commercial or financial relationships that could be construed as a potential conflict of interest.

## Supplementary Material

Supplemental Figure 1 Melting curves of randomly selected circRNAs for qPCR validation. All the melting curves were demonstrated as single peaks.

Supplemental Figure 2 **(A)** CircRNA-miRNA network analysis. The top 5 B-SPZ up-regulated circRNAs localized in head sperm and predicted miRNAs were selected to generate a network map. The circRNA-miRNA network was constructed using bioinformatics online programs (starBase, circBase, TargetScan, miRBase, Cytoscape). **(B)** The top 5 B-SPZ down-regulated circRNAs localized in head sperm and predicted miRNAs were selected to generate a network map. The circRNA-miRNA network was constructed using bioinformatics online programs (starBase, circBase, TargetScan, miRBase, Cytoscape). The symbols of hexagon, oval and rectangle represent circ-RNA, miRNA, and mRNA target, respectively. The continue arrow indicates the tethering activity of circRNA toward miRNA while the dotted arrow indicates the inhibitory activity of miRNA toward mRNA target.
