# Supplementary figures and images for: Expression Patterns of Circular RNAs in High Quality and Poor Quality Human Spermatozoa
Source: Front Endocrinol (Lausanne). 2019 Jul 3;10:435. doi: 10.3389/fendo.2019.00435 (PMC6626923; doi:10.3389/fendo.2019.00435)

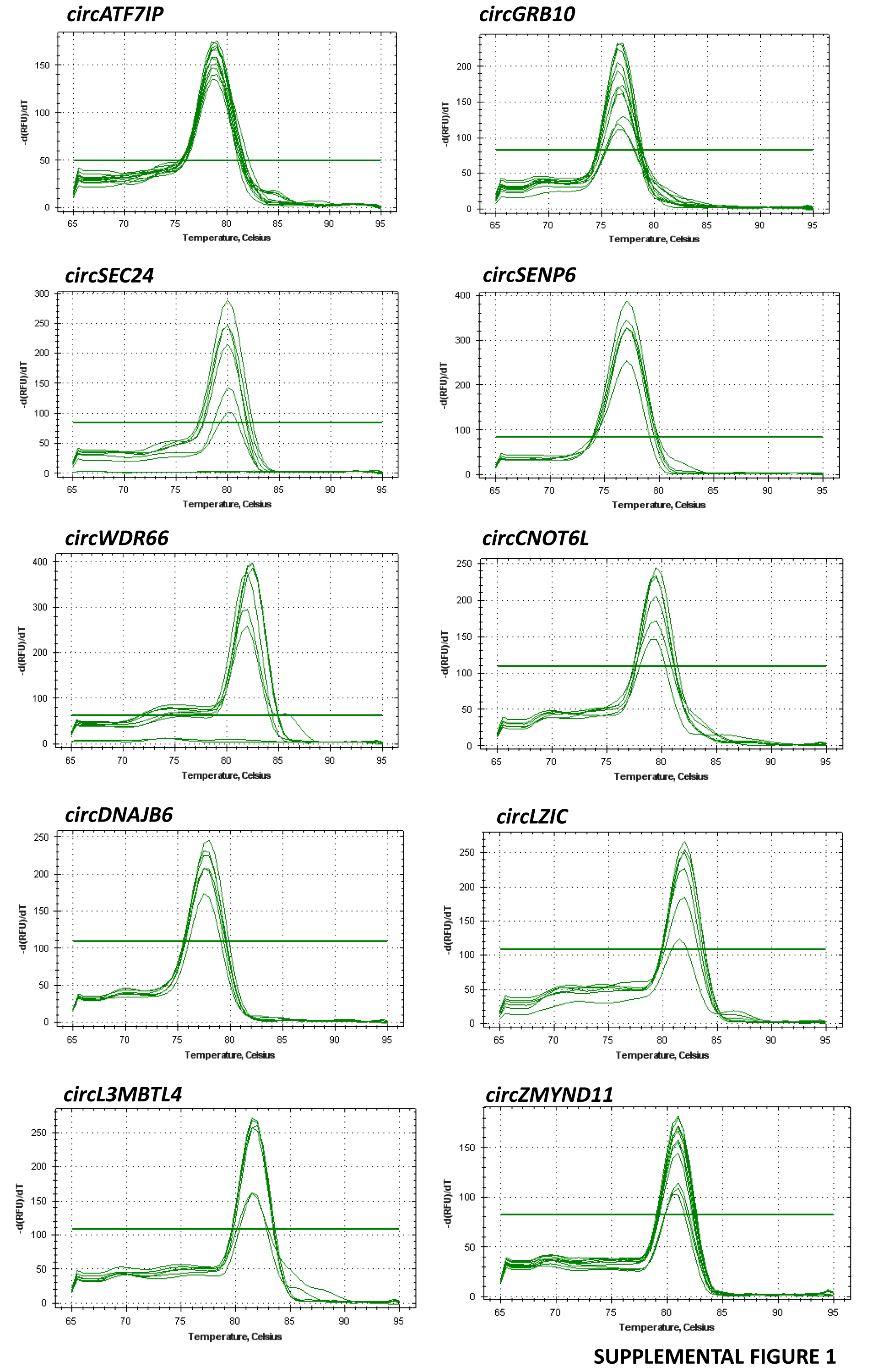

Supplement: Supplemental Figure 1 — Melting curves of randomly selected circRNAs for qPCR validation. All the melting curves were demonstrated as single peaks. [file Image_1.TIF]

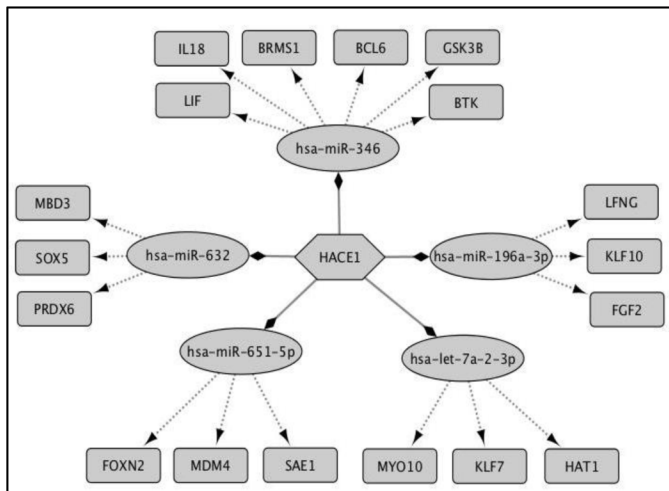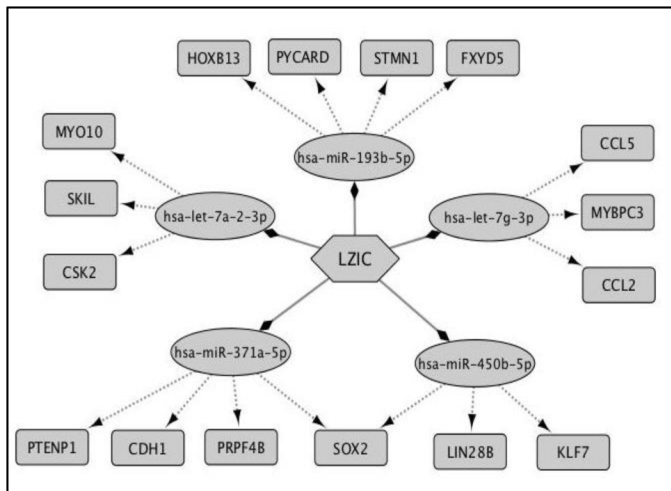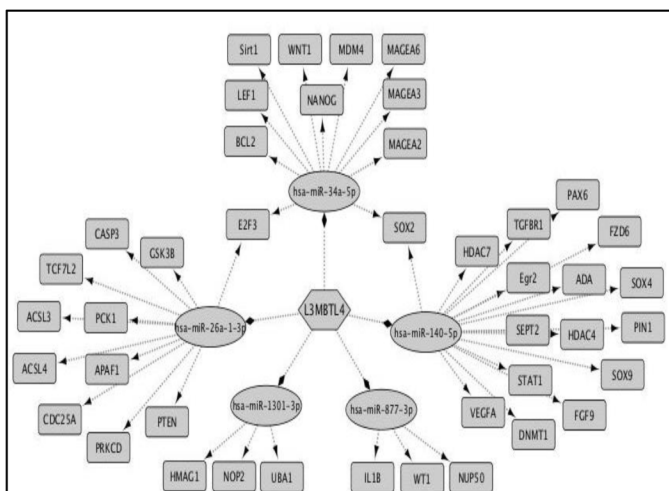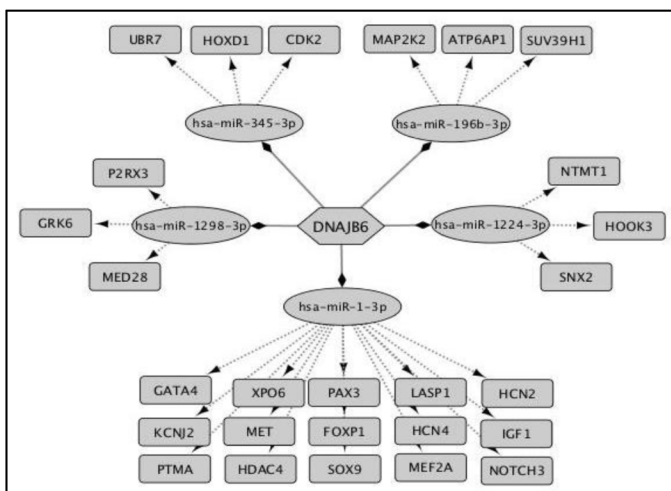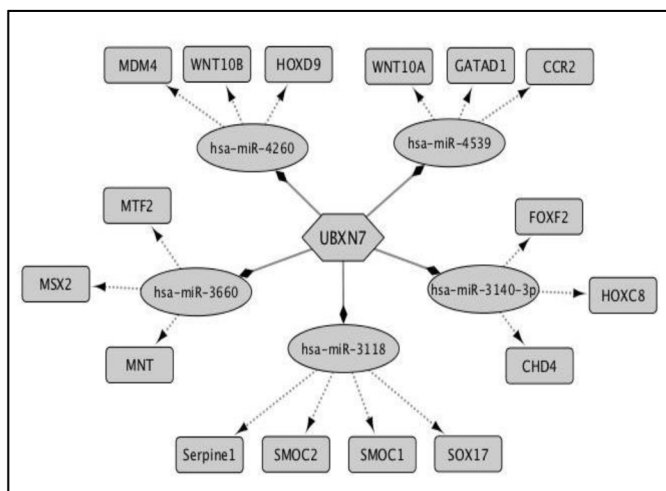

SUPPLEMENTAL FIGURE 2B

Supplement: Supplemental Figure 2 — (A) CircRNA-miRNA network analysis. The top 5 B-SPZ up-regulated circRNAs localized in head sperm and predicted miRNAs were selected to generate a network map. The circRNA-miRNA network was constructed using bioinformatics online programs (starBase, circBase, TargetScan, miRBase, Cytoscape). (B) The top 5 B-SPZ down-regulated circRNAs localized in head sperm and predicted miRNAs were selected to generate a network map. The circRNA-miRNA network was constructed using bioinformatics online programs (starBase, circBase, TargetScan, miRBase, Cytoscape). The symbols of hexagon, oval and rectangle represent circ-RNA, miRNA, and mRNA target, respectively. The continue arrow indicates the tethering activity of circRNA toward miRNA while the dotted arrow indicates the inhibitory activity of miRNA toward mRNA target. [file Image_2.pdf]
